# Assessing the Link Between Homeownership and Health in Canada: Evidence From the 2021 Canadian Housing Survey

**DOI:** 10.1111/cars.70041

**Published:** 2026-05-13

**Authors:** Min Zhou

**Affiliations:** ^1^ Department of Sociology University of Victoria Victoria British Columbia Canada

## Abstract

This study proposes a theoretical model that integrates important mediating and moderating effects to explain whether and how homeownership is potentially linked to health in today's Canadian society. This integrative model examines (1) whether the link between homeownership and health is moderated by housing cost (especially housing unaffordability) and (2) whether the homeownership–health link can be explained by the three mechanisms represented by three satisfaction perceptions—housing satisfaction, community satisfaction and life satisfaction—that stress homeownership's material, social and symbolic values, respectively. Guided by the theoretical model, the analyses of the 2021 Canadian Housing Survey data generate important insights. First, there is clear evidence that homeownership is significantly related to better health in Canada and that this link is not diminished by unaffordability. Second, homeownership is not directly linked with health, and the revealed link can be attributed to the three mechanisms. These underlying mechanisms, supported by empirical evidence, can explain how homeownership is related to better health and shed light on how housing inequality may translate into health disparity.

## Résumé

Cette étude propose un modèle théorique intégrant d'importants effets médiateurs et modérateurs afin d'expliquer si et comment l'accession à la propriété est potentiellement liée à la santé dans la société canadienne actuelle. Ce modèle intégratif examine (1) si le lien entre l'accession à la propriété et la santé est modéré par le coût du logement (en particulier son inabordabilité) et (2) si ce lien peut s'expliquer par trois mécanismes représentés par trois perceptions de satisfaction—satisfaction à l’égard du logement, satisfaction à l’égard de la communauté et satisfaction à l’égard de la vie—qui mettent respectivement l'accent sur les valeurs matérielles, sociales et symboliques de l'accession à la propriété. Guidées par le modèle théorique, les analyses des données de l'Enquête sur le logement au Canada de 2021 fournissent des informations importantes. Premièrement, il existe des preuves claires que l'accession à la propriété est significativement liée à une meilleure santé au Canada et que ce lien n'est pas atténué par l'inaccessibilité financière. Deuxièmement, l'accession à la propriété n'est pas directement liée à la santé, et le lien mis en évidence peut être attribué aux trois mécanismes. Ces mécanismes sous‐jacents, étayés par des données empiriques, permettent d'expliquer comment l'accession à la propriété est liée à une meilleure santé et de mieux comprendre comment les inégalités en matière de logement peuvent se traduire par des disparités en matière de santé.

## 1 Introduction

The right to adequate housing was considered a fundamental human right by the United Nations Committee on Economic, Social and Cultural Rights in 1991 and was recognized as a basic prerequisite for health according to the World Health Organization's Ottawa Charter for Health Promotion in 1986. For sociologists, the study of housing has transitioned from a specialized sub‐field to a core analytical framework for understanding the reproduction of social inequality in contemporary societies (Bartram and Brown‐Saracino 2025; McCabe 2016). While sociology has focused for decades on the workplace as the primary site of class formation, a burgeoning literature argues that housing, and specifically homeownership, represents an independent and often decisive axis of social stratification (Ruonavaara 2025). Housing is not only a physical shelter but also has profound influences on many aspects of our lives, including health (Clapham 2010; Hopton and Hunt 1996; Howden‐Chapman et al. 2023; Shaw 2004). There is a growing body of evidence from many societies that supports the influence of housing on health outcomes (Bentley et al. 2016; Swope and Hernández 2019). Adequate housing is associated with better health, while poor housing is found to be associated with infectious and chronic diseases, injuries, poor nutrition and mental disorders (Krieger and Higgins 2002; Shaw 2004).

Housing tenure is ‘frequently placed at the center of the housing and health relationship’ (Bentley et al. 2016, 210). Stable housing tenure, especially homeownership, is normally considered to be beneficial for health (Singh et al. 2019; Swope and Hernández 2019; Tranter and Donoghue 2017). However, there are several questions that remain understudied regarding the homeownership–health relationship in today's Canadian society. Despite the increasing awareness about housing crises experienced by many Canadians (Choi and Soave 2025; Tretter and Heyman 2022; Zhu et al. 2023), there has been very scarce research on how housing tenure embodied by homeownership is related to health among Canadians, and scholars have long been calling for empirical research in the Canadian context (Bryant 2003; Michel 2023). Specifically, this study is intended to fill two important gaps in our existing knowledge about the homeownership–health connections in Canada. First, given deteriorating housing affordability (Choi and Ramaj 2024; Choi and Soave 2025; Randle et al. 2021) and increasingly unattainable homeownership in today's Canada (Zhu et al. 2024; Zhu et al. 2023), owning a home may become a source of strain and distress and is increasingly out of reach for many Canadians. It remains an open question as to whether homeownership is still positively related to health and whether and to what degree housing unaffordability reduces or even offsets the positive homeownership–health link. Second, it remains unexplored how homeownership translates into better health in the Canadian context. Drawing upon a variety of sociological and social psychology studies on housing and health, this study also aims to reveal and assess the mechanisms through which homeownership relates to better health. Taken together, this study attempts to address the following research questions:

1. Is homeownership positively related to health in Canada?
2. Is the homeownership–health relationship affected by housing cost, especially housing unaffordability?
3. What are the mechanisms that can account for the homeownership–health relationship?

Because homeownership represents a key aspect of housing inequality in a society (Arundel and Ronald 2021; James et al. 2024), the investigation into the mechanisms underlying the homeownership–health link will contribute to a better understanding of the role played by housing inequality in shaping health disparity in today's Canada.

## 2 The Positive Link Between Homeownership and Health

There has been a long sociological tradition that highlights the importance of homeownership as a mechanism of social stratification. While Marxist analysis traditionally focuses on the ownership of productive capital, Weber (1946) defines class as a ‘market situation’ determined by one's ability to command assets, including property. Following Weber, sociologists such as Couper and Brindley (1975) have promoted the theory of ‘housing classes’, arguing that access to the ‘means of housing’ is a class‐forming factor in its own right. More recently, sociologists (Adkins et al. 2020) have reconfirmed the growing importance of housing in today's society, arguing that in a context of wage stagnation and house‐price inflation, property ownership may have taken precedence over employment in determining many aspects of one's experience and life chances.

Aligned with and influenced by this sociological tradition that highlights the importance of homeownership as a key determinant of one's social life, homeownership has attracted increasing academic interest as an important determinant of health (Garrison and Pollack 2018; Shaw 2004; Swope and Hernandez 2019). In earlier literature, the relationship between homeownership and health outcomes was not taken seriously or was taken for granted because homeownership was simply considered a proxy for socioeconomic status (SES), such as wealth and income that are necessary to buy a home (Shaw 2004). Individuals with higher SES commonly have better health. Homeownership is considered to be a transmission pathway by which SES affects health (Angel and Bittschi 2019), and the positive connection between homeownership and health would disappear once major SES indicators are controlled. However, more recent studies have realized the presence of an independent relationship between homeownership and health. Owning one's home provides important physical, financial and emotional utilities that generate additional benefits for health (Clapham 2005; Swope and Hernandez 2019). Renters or public housing tenants are found to experience more stress and show poorer health, compared with homeowners (Clair et al. 2016; Mason et al. 2013; Tranter and Donoghue 2017). Homeownership cannot be simply reduced to an indicator of SES.

The positive homeownership–health link may not always be positive, though (Shaw 2004), and not all homeownership is the same. Owning or buying a home can also be a source of stress and psychosocial burden, especially in a society that faces a housing crisis and high housing prices. Canada is considered to have a housing unaffordability crisis, with about one in five Canadian households spending 30% (the most commonly used threshold for measuring unaffordability) or more of their income on housing in 2021 (Statistics Canada 2022a, b). Canada has one of the most unaffordable housing markets and ranks high in the house‐price‐to‐income ratio among all OECD countries (Zhu et al. 2023). Hence, I first empirically assess the presence or absence of the positive homeownership–health link in today's Canada after other factors, including SES characteristics, are accounted for. Although I hypothesize a positive link, it remains an open question regarding the homeownership–health link in today's Canada.

Hypothesis 1 There is a significantly positive link between homeownership and health.

## 3 Moderating the Homeownership–Health Link: Housing Cost and Unaffordability

I next look into the potential conditioning effect of housing costs and unaffordability on the homeownership–health relationship. The existing literature sometimes suggests that the positive link between health and homeownership can be contingent on housing costs because high housing costs, housing unaffordability in particular, may reduce or even offset the positive homeownership–health link. Homeownership could be detrimental to health if it comes at the expense of high housing costs, especially if homeowners face housing unaffordability (Evans et al. 2003; Nettleton and Burrows 2000). The housing‐expenditure‐to‐income ratio (i.e., housing expenditures as a proportion of household income) is most commonly used to measure housing costs (Bentley et al. 2016; Hulchanski 1995). If individuals must spend a large portion of their household income on housing, they face housing unaffordability.

Higher housing costs imply that the individual has few resources and more difficulties in meeting their non‐housing needs. The financial strain of high housing costs gives rise to a tension between housing and non‐housing needs. When housing costs are high, homeowners are less able to pursue health‐promoting necessities such as a healthy diet, healthcare products and services and maintenance of social networks (Chung et al. 2020; Hernandez 2016; Meltzer and Schwartz 2016; Pollack et al. 2010). High housing costs also induce stress directly, such as the stress associated with long‐term debts and the stress of securing employment and income sources (Fletcher et al. 2009; Frank et al. 2006; King 2018). This conditional effect from housing costs may be especially salient for individuals experiencing housing unaffordability (Bentley et al. 2019; Lynch et al. 1997; Pollack et al. 2010). Homeowners facing housing unaffordability are more likely to experience potential mortgage delinquency or foreclosure and are thus at a higher risk for adverse health outcomes, including distress, anxiety, depression, chronic diseases and substance use (Alley et al. 2011; Burgard et al. 2012; Cannuscio et al. 2012; Tsai 2015), all of which reduce or even offset the link between owning a home and better health. In light of this line of literature, I assess the possible conditioning effect of housing costs and unaffordability and examine whether the connection between homeownership and better health would be reduced by housing costs and unaffordability in Canadian society.

Hypothesis 2 High housing costs, housing unaffordability in particular, reduce the positive link between homeownership and health.

## 4 Mediating the Homeownership–Health Link: Three Mechanisms

In order to shed light on how homeownership is actually related to better health, I identify three potential mechanisms. The three mechanisms stress the material, social and symbolic values of homeownership, respectively, and highlight how homeownership promotes housing satisfaction, community satisfaction and life satisfaction, respectively, which in turn contribute to better health.

### 4.1 Mediating Pathway 1: Homeownership → Perceived Better Housing Conditions → Better Health

First, homeownership is associated with more satisfactory housing conditions and provides a more stable and reliable shelter to live one's life. Homeownership brings about residential stability and less stress of being forced to relocate (Bentley et al. 2016). Homeowners generally demonstrate a greater ability to remain in their homes without worrying about losing their homes (Swope and Hernández 2019). Homeowners are also better able to choose housing that meets their needs and preferences, and privately owned housing is more likely to be located in more desirable neighbourhoods. In contrast, renters and public housing dwellers tend to live in less satisfactory housing environments, such as poor internal housing conditions and unhealthy and less safe areas (Macintyre et al. 2003). Homeowners are more willing and have more incentive to maintain and improve their homes according to personal preferences and health considerations, leading to better well‐being and health (Dietz and Haurin 2003; Green 2001). Homeowners can make structural adjustments to their dwellings and improve housing quality (Haurin et al. 2002; Thomson et al. 2013). People who rent their homes may have limited agency for housing maintenance or improvement. As a result, it is found that in many societies, homeowners are generally more satisfied with their housing situations (Elsinga and Hoekstra 2005), and more satisfactory housing brings about better physical and mental health (Swope and Hernández 2019; Zhu and Holden 2023).

Hypothesis 3 Homeownership increases one's housing satisfaction, which is related to better health.

### 4.2 Mediating Pathway 2: Homeownership → Perceived Better Sense of Community → Better Health

Second, homeownership is related to better health thanks to its social values and connection with social capital. Compared with renters, homeowners are usually better integrated into local communities. They have more motivation to engage in community building and socialize with their neighbours. Homeowners are found to have increased participation in community organizations (McCabe 2013), neighbourhood or block socialization (Manturuk et al. 2012) and local political activities (Yoder 2020). Homeowners are also found to have increased social ties to other homeowners in ones’ neighbourhood than renters (Manturuk et al. 2010). The improved social capital as a result of homeownership leads to greater attachment to the community (Manturuk et al. 2012) and improves homeowners’ social life and general well‐being (DiPasquale and Glaeser 1999; Engelhardt et al. 2010). This better social integration and greater social capital benefit health both directly and indirectly through more familiarity with and access to local health resources and community facilities. In contrast, renters may experience deleterious health effects associated with social isolation, lack of neighbourhood cohesion and community disconnectedness (Rohe et al. 2002).

Hypothesis 4 Homeownership increases one's sense of belonging to the local community, which is related to better health.

### 4.3 Mediating Pathway 3: Homeownership → Perceived Better Life Satisfaction → Better Health

Third, homeownership has a beneficial impact on health, also thanks to its symbolic values. Giddens (1991) argues that the home serves as a secure base for identity construction and a site where individuals gain ‘freedom from’ external scrutiny. Owning one's home provides the individual with important symbolic values that are well captured by such theories as ontological security (Dupuis and Thorns 1998; Hiscock et al. 2001), the norm or ideology of homeownership (McKee et al. 2017; Vassenden 2014) and the ‘nation of homeowners’ thesis (Saunders 1990). According to these theories, homeownership is socially and culturally constructed as a dominant norm and ideology in a society, and it confers symbolic meanings such as greater senses of security, achievement and social status, which are important underpinnings of higher life satisfaction. Owning a home offers a stronger sense of control, self‐esteem and empowerment, which all contribute to better satisfaction with life in general. This control over one's living space and the security of tenure create a generalized sense of control over important life events (Manturuk 2012; Rohe and Basolo 1997). People who become homeowners experience a greater sense of self‐esteem as they accomplish the goal of purchasing a home (Rossi and Weber 1996), often considered one of the greatest investments in their lives. The sense of control and that of self‐esteem lead to a sense of empowerment. Having successfully completed the difficult task and ‘dream’ of purchasing a home, homeowners may feel more empowered to take on other important tasks in life (Kleinhans and Elsinga 2010). In a society where the norm of homeownership is pervasive, homeownership is widely considered a main capital form of social standing and a standard for moral judgements of worth against those unable to access homeownership (McKee et al. 2017; Vassenden 2014). Taken together, owning a home provides symbolic advantages in the form of strengthened senses of control, autonomy, status and empowerment, and all these positive psychological effects form an important basis for satisfaction with one's own life (Rohe and Stegman 1994) and thus contribute to better well‐being (Manturuk 2012).

Hypothesis 5 Homeownership increases one's life satisfaction, which is related to better health.

## 5 Summary of Research Hypotheses

**FIGURE 1:**
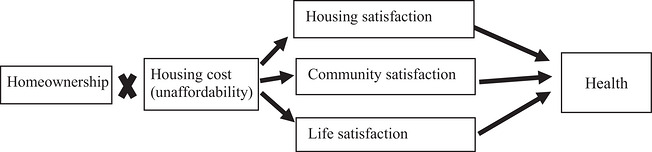
The hypothesized model of the homeownership–health relationship.

Figure 1 below summarizes the five hypotheses and visualizes the hypothesized theoretical model consisting of the homeownership–health relationship, the possible conditioning effect from housing costs (or unaffordability) and the three mediating mechanisms. This model will both guide and be assessed by the empirical analyses below.

## 6 Data and Methods

### 6.1 Data

To examine the five research hypotheses regarding the homeownership–health relationship in today's Canadian society, I analyse a large dataset from the 2021 Canadian Housing Survey (CHS). The 2021 CHS was conducted in all 10 provinces of Canada and three territories during the period from January to June 2021. The total sample size is 40,988. This survey provides information on how Canadians feel about their housing and how housing affects them (Statistics Canada 2021). It contains information on Canadians’ housing needs, housing tenure and characteristics, demographic and socioeconomic characteristics and self‐assessed health. According to Statistics Canada (2021, 5), ‘whenever possible, the survey was completed by the household member with the most knowledge of the household's housing situation. In all cases, this person was aged 15 years or older’.

The 2021 CHS strictly followed rigorous multi‐stage stratified random sampling and was largely nationally representative (Statistics Canada 2021). Specifically, its target population was the whole Canadian population, consisting of all 10 provinces and the territorial capitals of Whitehorse, Yellowknife and Iqaluit, excluding residents of institutions, members of the Canadian Forces living in military camps and people living on reserves and other Indigenous settlements. The survey covered about 98% of the population in the 10 provinces. Nevertheless, data collection in the three territories was limited to their respective capitals for operational reasons. Hence, for the three territories, the 2021 CHS was only representative of the population living in their capital cities. Taken together, the 2021 CHS provides high‐quality and the latest available data to assess the hypothesized model on the homeownership–health relationship. More information about the 2021 CHS can be found at this Statistics Canada webpage https://www23.statcan.gc.ca/imdb/p2SV.pl?Function=getSurvey&Id=1405275.

Statistical estimation of population characteristics using survey data is based on the premise that each sampled unit represents, in addition to itself, a certain number of non‐sampled units in the population. The survey design weight was calculated and provided by the 2021 CHS for each sampled unit based on its probability of selection to indicate the number of units within the population that the unit represents. All statistics and estimated models below are weighted according to the survey design weight so that the results are representative of the Canadian population from which the sample was drawn.

### 6.2 Dependent Variable

**FIGURE 2:**
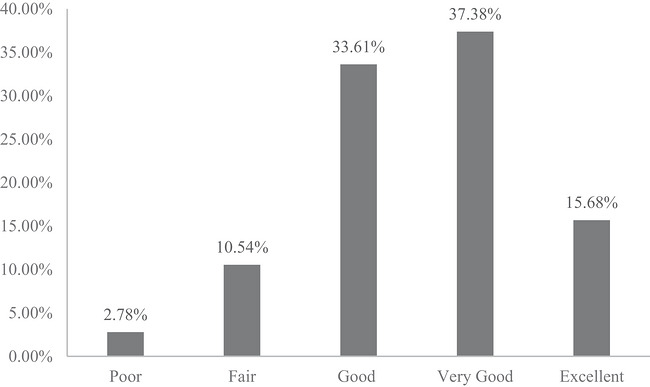
Overall distribution of self‐assessed health (survey weight–adjusted).

Self‐assessed health is used to capture individuals’ overall health status. I use self‐assessed general health based on the survey question ‘In general, how is your health?’ The responses are on a five‐point scale, including excellent, very good, good, fair and poor. I reverse‐coded the original scale used in the survey so that a greater number indicates better general health, with 1 indicating *poor health* and 5 *excellent health*. Self‐assessed health is a straightforward survey instrument to measure health, and more importantly, it is a reliable and commonly used assessment of individuals’ overall health (Benyamini 2011). Growing evidence has found it to be an effective measure of personal health status (McCallum et al. 1994; Wu et al. 2013) and a reliable predictor of major health outcomes, disease risks and mortality (Benyamini and Idler 1999; DeSalvo et al. 2006; Idler and Benyamini 1997). Figure 2 displays the distribution of self‐assessed health among the Canadian population. There are large variations in Canadians’ self‐assessed health, which will be analysed in the following analyses.

### 6.3 Explanatory Variables

Homeownership is captured by the question of whether a dwelling is owned by a member of the respondent's household, including themselves. It is a binary variable that identifies owning the home as 1 and not owning the home as 0. In the data, 67.9% of Canadian households own their homes, while 32.1% do not own. These numbers are consistent with recent homeownership rates reported by Statistics Canada (2022a, b). For example, the homeownership rate was reported to be 69.0% in 2011 and 66.5% in 2021 in Canada (Statistics Canada 2022a).

The three mediating variables include three perceptions about housing, the local community and overall life, respectively. I recoded the original survey data so that for all three variables, a higher score indicates a higher degree of satisfaction. Housing satisfaction is measured by the survey question ‘How satisfied are you with your dwelling?’ and the responses are on a three‐point scale, including dissatisfied (1), neutral (2) and satisfied (3).^1^ Community satisfaction is gauged by the survey question ‘How would you describe your sense of belonging to your local community?’ The responses are on a four‐point scale ranging from very weak (1), somewhat weak (2), somewhat strong (3) and very strong (4). Life satisfaction is captured by the survey question ‘How do you feel about your life as a whole right now?’ The responses are measured on a nine‐point scale with 1 denoting *very dissatisfied* and 9 *very satisfied*.^2^

Housing cost is calculated as the housing‐cost‐to‐income ratio, which refers to the proportion of the total household income spent on housing costs (i.e., rent or mortgage payments). The housing‐cost‐to‐income ratio is calculated by dividing the average monthly housing costs by the average monthly total household income. It is measured in the 2021 CHS data as a four‐category scale ranging from 1 (spending less than 30% of income on housing costs), 2 (spending 30% to less than 50% of income on housing costs), 3 (spending 50% to less than 100% of income on housing costs), to 4 (spending over 100% of income on housing costs).

There is no standard definition for housing unaffordability, but many scholars (Bentley et al. 2016; Hulchanski 1995; Schwartz and Wilson 2008), the Canada Mortgage and Housing Corporation (2018) and Statistics Canada (2022a, b) all define housing unaffordability as housing costs that are greater than 30% of the household's income. The threshold is based on the idea that if a family spends more than 30%, they will not have enough ‘residual income’ left for other non‐discretionary essentials such as food, healthcare, transportation and clothing (Herbert et al. 2018). People who have housing expenditures that take up 30% or more of their household income are categorized as facing housing unaffordability. The housing unaffordability variable is binary, with 1 indicating *housing unaffordability* and 0 as *being affordable*. In the data used here, the percentage of housing unaffordability is 19.6%, which is largely consistent with the number (20.9%) from the 2021 Canadian Census (Statistics Canada 2022a).

### 6.4 Control Variables

It is necessary to adjust for SES, such as education and household income, because wealthier and better educated individuals tend not only to have a higher likelihood of owning a home, but also better health (Baker et al. 2013). I also control for basic sociodemographic variables such as gender (Bentley et al. 2012; Park et al. 2022), age (Connolly 2012), racialized group status (Friedman and Rosenbaum 2004; Haan et al. 2024; Ortiz and Zimmerman 2013) and immigration status (Montazer 2022; Yu and Haan 2012), because these variables are also potential confounders and may be related to both homeownership and health. The 2021 CHS coded respondents’ age into four groups including ‘under 18’, ‘18–29’, ‘30–64’ and ‘65 and above’, so age is measured as a set of dummy variables including ‘18–29’, ‘30–64’ and ‘65 and above’, with ‘under 18’ as the reference category. In particular, there are 2892 respondents below 18 years old. I also tried limiting all data analyses to respondents who are 18 years old or older, which resulted in a slightly reduced sample size of 38,096, and found all the findings substantively the same. Gender was measured as male and female in the 2021 CHS, so the gender variable used here is binary, with 1 indicating *male* and 0 *female*. The racialized group is a binary variable, and the survey measures it as ‘the presence of a visible minority household member (excluding persons in the household who are unrelated to the respondent)’ which is coded as 1. Immigration status is measured as binary, which indicates whether the respondent is an immigrant. A non‐permanent resident is coded as 1, and a non‐immigrant (a Canadian‐born citizen) coded as 0. The education variable measures the highest level of education that the respondent has completed. It was coded as seven educational groups in the survey, including ‘less than high school diploma or its equivalent’, ‘high school diploma or an equivalency certificate’, ‘trade certificate or diploma’, ‘college, CEGEP or other non‐university certificate’, ‘university certificate or diploma below bachelor's level’, ‘bachelor's degree’ and university certificate, diploma, degree above the bachelor's’. I measure education as a set of dummy variables with ‘less than high school diploma or its equivalent’ as the reference group. Household income is calculated as the sum (in Canadian dollars) of the total income of all members of the respondent's household.

I further control for key aspects of housing characteristics, including residential length, dwelling type, the number of bedrooms, housing conditions and housing environment (neighbourhood) in order to ensure the effects of homeownership on health and various satisfaction perceptions are not confounded by other aspects of housing (Carson et al. 2010; MacIsaac 2024; Swope and Hernández 2019). Residential length is measured by how long the respondent has been living in their current dwelling. The possible responses are on a four‐point scale, including ‘less than 2 years’ coded as 1, ‘2 years to less than 5 years’ as 2, ‘5 years to less than 10 years’ as 3 and ‘10 or more years (including always lived here)’ as 4. Hence, a greater value indicates living in the current dwelling for a longer time.

Dwelling type and the number of bedrooms capture dwelling characteristics. Dwelling type is measured in the survey by seven categories that represent ‘single‐detached house’, ‘semi‐detached house’, ‘row house’, ‘apartment or flat in a duplex’, ‘apartment in a building that has five or more storeys’, ‘apartment in a building that has fewer than five storeys’ and ‘other’. I create a set of six dummy variables with ‘single‐detached house’ as the omitted and reference category. The number of bedrooms is measured by four categories in the survey, including ‘one or fewer’, ‘two’, ‘three’ and ‘four or more’. I use three dummy variables with ‘one or fewer’ as the omitted and reference category.

Housing conditions are captured by two variables in the survey. The first one, repair needed, is measured by the survey question ‘Is this dwelling in need of any repairs?’. It is a binary variable with 1 indicating the dwelling in need of repairs and 0 not. The second one, dwelling problems, is measured by a series of four questions regarding some of the common issues related to the dwelling's condition: ‘In the past 12 months, have you experienced any of the following issues in your dwelling? (1) Patches of mould or mildew; (2) infestations of unwanted pests; (3) undrinkable water; (4) regular poor indoor air quality’. Each of the four questions is a yes or no question, with *yes* coded as 1 and *no* as 0. I then add up the respondents’ answers to the four questions to generate a composite variable, dwelling problems, representing the dwelling's condition. The variable is on a five‐point scale ranging from 0 to 4. A larger value indicates more problems with the dwelling, suggesting a poorer housing condition. I also tried using the four questions as four separate control variables in the regression analyses, which did not substantively change the findings.

Housing environment is gauged by a set of survey items about some of the major issues in the neighbourhood where the dwelling is located. The respondent is asked, ‘In your neighborhood, how much of a problem are the following issues? (1) Noisy neighbors or loud parties; (2) people hanging around on the streets; (3) garbage or litter lying around; (4) vandalism, graffiti and other deliberate damage to property or vehicles; (5) people being attacked or harassed because of their skin color, ethnic origin or religion; (6) people using or dealing drugs; (7) people being drunk or rowdy in public places; (8) abandoned buildings; (9) smog or air pollution’. For each of the nine questions, the answers are on a four‐point scale ranging from 0 (not a problem), 1 (a small problem), 2 (a moderate problem) to 3 (a big problem). I add up the respondents’ answers to the nine questions to create the composite ‘neighbourhood problems’ variable that ranges from 0 to 27, with a greater value indicating more problems in the neighbourhood and thus a poorer housing environment. I also tried including the nine questions as nine separate control variables in the regression analyses, which did not substantively change the findings.

Table 1 summarizes key descriptive statistics for the variables used in the analyses.

**TABLE 1:** Descriptive statistics for all variables used in the analyses.

Variables	Mean	Std. dev.	Minimum	Maximum
Self‐assessed health	3.465	1.016	1	5
Homeownership	0.679	0.467	0	1
Housing satisfaction	2.542	0.701	1	3
Community satisfaction	2.669	0.814	1	4
Life satisfaction	6.452	2.024	1	9
Housing cost	1.278	0.629	1	4
Housing unaffordability	0.196	0.397	0	1
Age groups:				
Under 18	0.085	0.278	0	1
18–29	0.263	0.440	0	1
30–64	0.378	0.485	0	1
65 and over	0.275	0.447	0	1
Gender (male)	0.510	0.500	0	1
Racialized group	0.237	0.425	0	1
Immigrant	0.281	0.450	0	1
Educational groups:				
Less than high school	0.061	0.238	0	1
High school	0.172	0.378	0	1
Trade certificate or diploma	0.080	0.271	0	1
College or non‐university certificate	0.209	0.406	0	1
University below bachelor's level	0.058	0.235	0	1
Bachelor's degree	0.247	0.431	0	1
University above the bachelor's	0.174	0.379	0	1
Household income (in thousands)	109.407	103.393	−60	1300
Residential length	3.028	1.091	1	4
Dwelling types:				
Single‐detached house	0.514	0.500	0	1
Semi‐detached house	0.043	0.203	0	1
Row house	0.059	0.235	0	1
Apartment in a duplex	0.045	0.207	0	1
Apartment (five or more storeys)	0.097	0.296	0	1
Apartment (fewer than five storeys)	0.173	0.379	0	1
Other	0.069	0.253	0	1
Number of bedrooms:				
One or fewer	0.133	0.340	0	1
Two	0.251	0.433	0	1
Three	0.343	0.475	0	1
Four or more	0.273	0.445	0	1
Repair needed	0.306	0.461	0	1
Dwelling problems	0.308	0.629	0	4
Neighbourhood problems	3.103	4.571	0	27

*Note*: The data are survey weight–adjusted so they are representative of the Canadian population.

### 6.5 Methods

The dependent variable, self‐assessed health, is ordinal on a five‐point scale, so I chose the ordinal logistic regression and the structural equation model (SEM) built upon the ordinal logistic regression to investigate the effects of homeownership, housing cost and unaffordability, the set of satisfaction perceptions, as well as the control variables, on health. Moreover, I need to test two types of relationships, moderation and mediation, stressed in the theoretical model. First, I estimate a series of ordinal logistic regression models that include the interaction term between homeownership and housing cost (or unaffordability) to assess whether and how housing cost (or unaffordability) conditions the effect of homeownership on health. A key assumption underlying the ordinal logistic regression is the proportional odds assumption, that is, the effects of the independent variables are largely consistent across all levels of the ordinal dependent variable. I conducted the likelihood‐ratio test of this assumption across response categories in the ordinal dependent variable. The test result was not statistically significant, indicating no violation of this proportional odds assumption.

Second, I employ the SEM to examine the mediating effects of the three satisfaction perceptions, respectively, on the homeownership–health relationship. The SEM can simultaneously estimate how homeownership directly and indirectly (through satisfaction perceptions) affects health (Acock 2013; Kline 2023). The three satisfaction perceptions are hypothesized to be the key mediating variables through which homeownership indirectly affects health. The SEM consists of four sub‐models with the three mediating variables (housing satisfaction, community satisfaction, life satisfaction) and self‐assessed health as the dependent variables, respectively. Because all four dependent variables are ordinal variables, the SEM is built upon four ordinal logistic regression models, and as a result, I choose the generalized SEM (GSEM) that can better accommodate ordinal dependent variables and produce better estimation (Acock 2013).

I utilize the Stata software (release 16) (StataCorp 2019) to estimate the ordinal logistic and GSEM models based on maximum likelihood estimation. For all estimated models, I use robust standard errors adjusting for clustering by cities or areas included in the 2021 CHS data, and base all inferential tests of regression coefficients on these robust standard errors. Pseudo *R* ^2^ (McFadden's *R* ^2^) is also reported for each estimated model. To assess the robustness of the mediating effects from the three satisfaction perceptions in the relationship between homeownership and health, I apply the Karlson–Holm–Breen (KHB) decomposition method (Breen et al. 2018, 2021; Karlson et al. 2012). In ordinal logistic models, changes in coefficients across nested models can sometimes reflect not only mediation but also the rescaling issue. The KHB method is capable of comparing coefficients across nested non‐linear probability models (such as ordinal logistic models used here) and separating the change in coefficients into the component attributable to mediation by the added variables and the component due to rescaling. In doing so, it ensures that the difference in the coefficients between nested models reflects actual mediating effects rather than statistical artefacts of rescaling. The KHB command in Stata is utilized to conduct this KHB decomposition.

## 7 Results

I first estimate a battery of ordinal logistic regression models to examine whether homeownership displays an overall positive relationship with health (Hypothesis 1) and whether this positive link is conditioned, especially reduced, by high housing cost and unaffordability (Hypothesis 2). The estimation results are presented in Table 2.

**TABLE 2:** Ordinal logistic regression of self‐assessed health.

Variable	Model 1	Model 2	Model 3	Model 4	Model 5
Homeownership	0.318^***^	0.360^***^	0.346^**^	0.294^***^	0.300^***^
	(0.066)	(0.041)	(0.126)	(0.066)	(0.072)
Housing cost		−0.166^***^	−0.147^*^		
		(0.025)	(0.058)		
Homeownership **×** Housing cost			−0.033		
			(0.082)		
Housing unaffordability				−0.289^***^	−0.276^**^
				(0.063)	(0.084)
Homeownership **×** Housing unaffordability					−0.023
					(0.123)
AIC	47,855.6	47,153.2	47,154.8	**47,151.4**	47,153.2
BIC	48,127.4	47,432.2	47,441.6	**47,430.5**	47,440.0
Pseudo‐*R* ^2^	0.131	0.135	0.135	0.135	0.135

*Notes*: (1) The data are survey weight–adjusted so they are representative of the Canadian population; (2) all models include controls for age groups, gender, the racialized group, immigration status, educational groups, household income, residential length, dwelling types, the number of bedrooms, repair needed, dwelling problems and neighbourhood problems; (3) numbers in parentheses are robust standard errors adjusting for clustering by cities or areas; (4) from two‐tailed tests; (5) in the ordinal regression model of self‐assessed health, four ‘intercepts’ or cut‐points are calculated which indicate where the latent variable is cut to make the five categories that we observe in the data of the self‐assessed health variable; they are not reported in the table to avoid clutter; (6) the AIC and BIC numbers for Model 4 are in bold as they are the smallest and thus suggest the best model fit.

^*^ *p* < 0.05; ^**^ *p* < 0.01; ^***^ *p* < 0.001.

Model 1 focuses on the variable of major interest, homeownership, while controlling for all demographic, socioeconomic and housing variables, including age groups, gender, the racialized group, immigration status, educational groups, household income, residential length, dwelling types, the number of bedrooms, repair needed, dwelling problems and neighbourhood problems. Homeownership displays a significant and positive relationship with health, net of all the influences from the other variables.

Model 2 includes homeownership and housing cost in addition to the control variables. Homeownership continues to show a significantly positive relationship with health, and the coefficient of housing cost is significantly negative. Higher housing cost is significantly associated with poorer health. Model 3 further incorporates the interaction term between homeownership and housing cost, and the interaction effect is not significant. While both homeownership and housing cost are significantly connected with health, there is no significant interaction effect between them. Hence, homeownership and housing cost operate independently in their respective relationships with health, and housing cost does not moderate the homeownership–health relationship.

Model 4 looks at the effect of housing unaffordability instead of housing cost. The negative coefficient of housing unaffordability is statistically significant, indicating that housing unaffordability has a significantly negative relationship with health. Consistent with the effect of housing cost observed in the previous models, it is found that housing unaffordability, a form of high housing cost, is negatively related to health. Nevertheless, according to Model 5, which further incorporates the interaction term between homeownership and housing unaffordability, there is no significant interaction effect. Taken together, although housing unaffordability is significantly related to poorer health, its effect does not interact with that of homeownership. Unaffordability does not significantly change the homeownership–health relationship.

**FIGURE 3:**
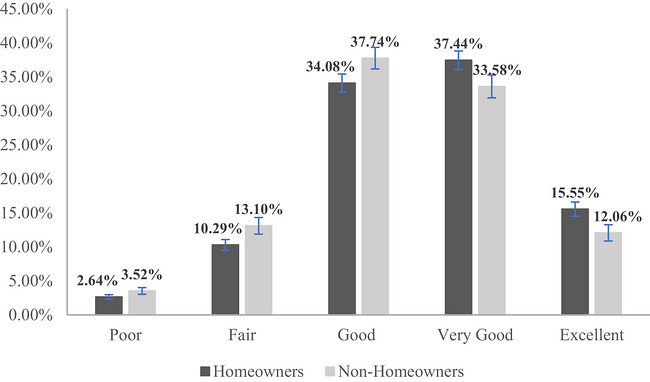
Effects of homeownership on self‐assessed health: predicted probabilities in each category of self‐assessed health (with 95% confidence intervals). *Note*: This figure is based on Model 4 that shows the best model fit among all models in Table 2, with all control variables held under control and at their respective mean values; error bars are 95% confidence intervals. [Colour figure can be viewed at wileyonlinelibrary.com]

Hence, the results revealed by the models support Hypothesis 1 about the positive homeownership–health relationship but provide no support for Hypothesis 2 about this positive relationship being conditional on housing cost or unaffordability. For each estimated model, I report the commonly used indicators for the goodness of model fit, the Akaike information criterion (AIC) and the Bayesian information criterion (BIC) (Akaike 1974; Raftery 1995). When a series of models is applied to the same data, the model with a smaller value of the AIC and BIC has a better model fit and can thus be considered a better model. Among the models in Table 2, Model 4 has the smallest AIC and BIC values, so it can be considered the one with the best model fit. Hence, based on the best‐fit Model 4, I calculate and visualize the significant relationship of homeownership with health, while holding all other variables under control and at their mean values. Figure 3 shows the predicted probabilities of falling into each health category for homeowners and non‐homeowners, respectively, with 95% confidence intervals displayed as error bars. It is worth noting that for each health category, I also test the statistical significance of the difference between homeowners and non‐homeowners. The difference in each health category is found to be statistically significant. The 95% confidence intervals for homeowners and non‐homeowners do not overlap in any of the five health categories, as shown in Figure 3. Hence, there is a statistically significant difference across all health statuses between homeowners and non‐homeowners. Specifically, homeowners are significantly more likely to have ‘very good’ and ‘excellent’ health, while non‐homeowners have a significantly higher chance of reporting ‘poor’, ‘fair’ or ‘good’ health. For example, owning a home alone is associated with an increase of 3.49 percentage points (from 12.06% to 15.55%) in one's chance of having excellent health, after all other variables have been accounted for. This difference of 3.49 percentage points is substantial, given the fact that it is attributable to homeownership alone, net of the influences from all other variables.

Guided by the scheme shown in Figure 1, I estimate two GSEMs, Models 6 and 7, which use housing cost and house unaffordability (the 30%‐cutoff measure), respectively. The estimated GSEMs include all demographic, socioeconomic and housing control variables, housing cost or unaffordability and homeownership, in addition to the mediating variables represented by the set of three satisfaction perceptions.

The estimated GSEM results are presented in Table 3. The whole modelling consists of four sub‐models—Sub‐model 1 with housing satisfaction as the dependent variable, Sub‐model 2 with community satisfaction as the dependent variable, Sub‐model 3 with life satisfaction as the dependent variable and Sub‐model 4 with self‐assessed health as the dependent variable. For the sake of clarity, although the four models are estimated simultaneously by the GSEM, I discuss their results separately.

**TABLE 3:** Generalized structural equation model (GSEM) of self‐assessed health.

(1) Model 6: GSEM using housing cost				
	Sub‐model 1	Sub‐model 2	Sub‐model 3	Sub‐model 4
	Housing satisfaction	Community satisfaction	Life satisfaction	Self‐assessed health
Homeownership	0.911^***^ (0.058)	0.351^***^ (0.052)	0.558^***^ (0.051)	0.096 (0.055)
Housing cost	0.001 (0.045)	−0.095^*^ (0.042)	−0.203^***^ (0.039)	−0.044 (0.043)
Housing satisfaction				0.184^***^ (0.042)
Community satisfaction				0.265^***^ (0.033)
Life satisfaction				0.343^***^ (0.016)
(2) Model 7: GSEM using housing unaffordability				
Homeownership	0.907^***^ (0.059)	0.351^***^ (0.053)	0.551^***^ (0.051)	0.091 (0.055)
Housing unaffordability	−0.039 (0.068)	−0.127^*^ (0.061)	−0.319^***^ (0.059)	−0.109 (0.063)
Housing satisfaction				0.185^***^ (0.042)
Community satisfaction				0.265^***^ (0.033)
Life satisfaction				0.343^***^ (0.016)

*Notes*: (1) The data are survey weight–adjusted so they are representative of the Canadian population; (2) all models include controls for age groups, gender, the racialized group, immigration status, educational groups, household income, residential length, dwelling types, the number of bedrooms, repair needed, dwelling problems and neighbourhood problems; (3) numbers in parentheses are robust standard errors adjusting for clustering by cities or areas; (4) from two‐tailed tests; (5) for all the four models which are all ordinal logistic regression models, the ‘intercepts’ or cut‐points are not reported in the table to avoid clutter.

^*^ *p* < 0.05; ^***^ *p* < 0.001.

Whether using housing cost (Model 6) or housing unaffordability (Model 7), the estimated results are substantively the same, so I discuss the two models together. For both models, their Sub‐models 1–3 estimate how homeownership contributes to housing satisfaction, community satisfaction and life satisfaction, respectively. Homeownership shows a significant effect in all three sub‐models. Specifically, individuals who own their homes are more likely to be satisfied with their housing conditions (Sub‐model 1), are more likely to feel a sense of belonging to the local community (Sub‐model 2) and are likely to be happy with their life overall (Sub‐model 3).

**FIGURE 4:**
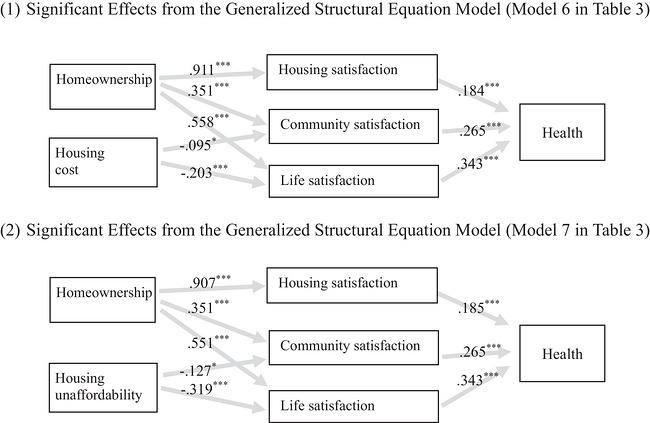
The moderating pathways in the homeownership–health relationship. (1) Significant effects from the generalized structural equation model (Model 6 in Table 3). (2) Significant effects from the generalized structural equation model (Model 7 in Table 3). *Note*: Both models include controls for age groups, gender, the racialized group, immigration status, educational groups, household income, residential length, dwelling types, the number of bedrooms, repair needed, dwelling problems and neighbourhood problems.

Sub‐model 4 assesses the relationships of homeownership, housing satisfaction, community satisfaction and life satisfaction with health. The three satisfaction variables all have significant effects, while the effect of homeownership disappears and becomes non‐significant. Specifically, those who are more satisfied with their housing conditions, local community and overall life tend to report better health. After the three satisfaction variables have been taken into account, the homeownership–health relationship is no longer significant. This result suggests that the significantly positive homeownership–health link observed in the previous models of Table 2 can be explained by the three mediating mechanisms. Figure 4 visualizes the mediating pathways and the indirect effects of homeownership revealed in the two GSEMs.

To check the robustness of the mediating effects, I further apply the KHB method in order to reveal to what degree the observed reduction in the coefficient of homeownership can be attributed to mediation by the three satisfaction variables as opposed to the rescaling issue. Two ordinal logistic models based on the KHB method, one using housing cost and the other using housing unaffordability, are estimated. According to the KHB decomposition, the inclusion of the three satisfaction variables reduces the coefficient of homeownership by 33.50% and 34.27% in the model that uses housing cost and in the model that uses housing unaffordability, respectively. The reduction attributable to the three satisfaction variables in both models is statistically significant and is considerable in size (well above 30%). Hence, after rescaling has been accounted for, the three satisfaction variables are still found to mediate the homeownership–health relationship. Over 30% of the difference in the coefficients between the models with and without the three satisfaction variables reflects actual mediating effects rather than rescaling artefacts.

How do these results bear out the proposed hypotheses? Hypotheses 3–5 about the mediating effects of housing satisfaction, community satisfaction and life satisfaction are all supported by the empirical evidence. The results lend support to these hypotheses regarding the indirect relationship of homeownership with health through housing satisfaction, community satisfaction, and life satisfaction. Homeownership contributes to the three satisfaction perceptions that promote better health. Once these mediating effects from the three perceptions have been taken into account, homeownership shows no direct relationship with health. Taken together, the three mediating mechanisms hypothesized in Hypotheses 3–5 account for the positive homeownership–health relationship.

## 8 Conclusion and Discussion

This study endeavours to reveal the relationship of homeownership with health in today's Canadian society, using the latest available nationally representative data. I propose a theoretical model that integrates important mediating and moderating effects to explain how homeownership is potentially linked to health. Building upon a wealth of existing literature, this integrative model examines (1) whether the link between homeownership and better health is moderated by housing cost, especially housing unaffordability, and (2) whether the positive homeownership–health link can be explained by the three mechanisms represented by the three satisfaction perceptions—housing satisfaction, community satisfaction and life satisfaction, respectively. In doing so, this study answers important questions regarding the health implications of homeownership, including the question of whether the positive homeownership–health link would be reduced or even offset by high housing costs (housing unaffordability, in particular) and the question of how exactly homeownership translates into better health and what mechanisms are at work in today's Canada where housing unaffordability is becoming increasingly severe (Choi and Ramaj 2024; Choi and Soave 2025; Randle et al. 2021) and homeownership is highly valued but increasingly unattainable (Zhu et al. 2024; Zhu et al. 2023).

Based on the empirical evidence here, it is found that homeownership is positively related to health and that this positive relationship is not diminished, let alone offset, by housing cost or housing unaffordability. While high housing cost and housing unaffordability are negatively related to people's health, they do not reduce the positive link between homeownership and health. On average, homeowners, even those facing affordability challenges, are consistently healthier than non‐homeowners. This finding gives support to the importance of homeownership in today's Canadian society and highlights the robust homeownership–health link against the backdrop of an increasingly unaffordable housing market. Homeownership and housing unaffordability are both related to health, but their relationships with health operate independently.

This study also identifies the three pathways through which homeownership is linked to better health. The three mechanisms that stress the material, social and symbolic values of homeownership, respectively, underpin the revealed homeownership–health nexus. First, homeownership provides people with more agency, freedom and motivations to alter and improve their dwellings according to personal preferences and health considerations (Dietz and Haurin 2003; Elsinga and Hoekstra 2005; Green 2001; Haurin et al. 2002; Thomson et al. 2013), which leads to more satisfactory housing conditions that elevate their well‐being and health. This ‘homeownership→housing satisfaction→better health’ mechanism finds empirical support and is a material pathway of homeownership leading to better health. Second, homeownership brings about better social integration and greater social capital (DiPasquale and Glaeser 1999; Engelhardt et al. 2010; Manturuk et al. 2010, Manturuk et al. 2012; McCabe 2013; Rohe et al. 2002; Yoder 2020). Homeowners are better integrated into local communities and are more motivated to participate in community activities. This greater social integration and enhanced social capital, embodied by greater community satisfaction, contribute to better health. The ‘homeownership→community satisfaction→better health’ mechanism is also supported by empirical evidence here, and this social pathway highlights the health benefit of social values embedded in homeownership. Third, as Canada is a ‘nation of home owners’ where homeownership is widely valued and considered to be the norm (Zhu et al. 2024; Zhu et al. 2023), there are socio‐culturally constructed symbolic values attached to homeownership (Dupuis and Thorns 1998; Hiscock et al. 2001; McKee et al. 2017; Saunders 1990; Vassenden 2014). Homeownership offers the individual a greater sense of security, control over life, achievement, dignity, social status and empowerment (Kleinhans and Elsinga 2010; Manturuk 2012; Rohe and Basolo 1997; Rossi and Weber 1996), thereby leading to greater life satisfaction and resulting in better well‐being and health. This ‘homeownership→life satisfaction→better health’ mechanism, also supported by empirical evidence here, reflects the symbolic value of homeownership that contributes to better health. Moreover, after the three mechanisms are accounted for, homeownership no longer shows a direct relationship with health. Therefore, the three mechanisms are successful in explaining why and how homeownership is linked to better health.

Taken together, the findings offer important insights into the homeownership–health link in today's Canadian society. Homeownership is not only a key aspect of housing inequality (Arundel and Ronald 2021; James et al. 2024), but, according to the findings here, it is also an important factor associated with health inequality in today's Canada. Homeownership itself does not have a direct relationship with health; the revealed homeownership–health link can be attributed to the three underlying mechanisms. These insights complement each other and provide us with a nuanced understanding of the relationship between homeownership and health in Canada. Practical and policy implications can be derived from these insights. To promote better health in today's Canadian society, on the one hand, in light of the positive homeownership–health link, social policies should aim to offer more support to potential home buyers and help them achieve homeownership through providing more accessible financial assistance and making housing prices more affordable. On the other hand, in light of the mechanisms that give rise to the homeownership–health link, we should promote measures that can help non‐homeowners, such as renters and public housing residents, benefit from the three mechanisms as much as homeowners. Considering the first moderating pathway, it would be helpful to establish policies that make it easy for non‐homeowners to alter their housing conditions in accordance with their personal preferences and health needs (so that they can benefit from the first ‘material’ mechanism). In light of the second moderating pathway, more measures should be put forward that help non‐homeowners better participate in community activities, make them feel more welcomed, and facilitate their integration into local communities (so that they can benefit from the second ‘social’ mechanism). In light of the third moderating pathway, it is also important to have policies that better protect non‐homeowners’ housing security and eliminate the stigma attached to renting or living in public housing (so that they can benefit from the third ‘symbolic’ mechanism). When working along these lines, we will be better able to extend the benefits associated with the three mechanisms to non‐homeowners in Canadian society, thereby reducing the housing‐related health disparity between homeowners and non‐homeowners.

Finally, although this study sheds important light on the homeownership–health relationship, I acknowledge and reflect on the limitations of this study. First, we should use caution when interpreting the findings and avoid making strong causal assertions. Given the cross‐sectional nature of the data, the analyses conducted here are not able to establish a definitive causal relationship between homeownership and better health. Potential endogeneity issues can be present, and the causal direction may not be exclusively one‐way from homeownership to better health. For example, the relationship can also be two‐way, with health also affecting homeownership. Healthier individuals may be more likely to achieve homeownership. Although the key socioeconomic variables controlled in the analyses are helpful, they cannot completely eliminate the possibility of endogeneity. In future studies, instrumental variables and panel data methods should be employed to help establish causality more definitively. Qualitative research, such as in‐depth interviews, can also further advance our understanding of the mechanisms that explain how homeownership gives rise to better health in practice. Second, although the 2021 CHS is a high‐quality national survey that provides rich housing‐related information, its data also impose some limitations on this study. For example, while the dependent variable, self‐assessed health, is generally considered a reliable measure of individuals’ overall health (Benyamini 2011; McCallum et al. 1994; Wu et al. 2013) and is commonly used in similar studies (Benyamini and Idler 1999; DeSalvo et al. 2006; Idler and Benyamini 1997), it can be prone to reporting biases related to age, gender, SES or cultural background (Groot 2000; Hunt and Bhopal 2004; Jylhä 2009). More specific and objective indicators, such as chronic illness, hospitalization and physical function status, should be included in future surveys. For operational reasons, in the 2021 CHS, data collection in the three territories is limited to their respective capitals and also does not cover people living on reserves and other Indigenous settlements. As a result, we should use caution and avoid generalizing the findings to the territorial northern and Indigenous populations. It is also worth mentioning that the 2021 CHS was conducted during the COVID‐19 pandemic, so this observed relationship between homeownership and health is inevitably affected by the pandemic. For example, the pandemic might have affected the desirability of homeownership. With more recent data becoming available, it would be interesting to reassess the key findings based on more recent data. It would also be worth applying the analyses to other societies in future research and exploring whether and to what extent the findings here are unique to Canada.

## Endnotes

1 In the original questionnaire, responses to the ‘housing satisfaction’ question are measured on a 0–10 scale. Due to the high concentration of responses in a few points on the scale, the final dataset provided by Statistics Canada classifies all responses into a 3‐point scale, including ‘dissatisfied’, ‘satisfied’ and ‘very satisfied’.

2 In the original questionnaire, responses to the ‘life satisfaction’ question are measured on a 0–10 scale. However, in the final dataset provided by Statistics Canada, responses are classified into a 9‐point scale as Statistics Canada combines the first three points (0, 1 and 2) into one point because of the small number of respondents choosing each of these three points.
